# Molecular epidemiology of drug resistant *Mycobacterium tuberculosis* in Africa: a systematic review

**DOI:** 10.1186/s12879-020-05031-5

**Published:** 2020-05-13

**Authors:** Namaunga Kasumu Chisompola, Elizabeth Maria Streicher, Chishala Miriam Kapambwe Muchemwa, Robin Mark Warren, Samantha Leigh Sampson

**Affiliations:** 1 DST/NRF Centre of Excellence for Biomedical Tuberculosis Research/South African Medical Research Council Centre for Tuberculosis Research, Division of Molecular Biology and Human Genetics, Faculty of Medicine and Health Sciences, Stellenbosch University, Cape Town, South Africa; 2 Department of Basic Medical Sciences, Michael Chilufya Sata School of Medicine, Copperbelt University, Ndola, Zambia

**Keywords:** *Mycobacterium tuberculosis*, Drug resistance, Africa, Molecular epidemiology

## Abstract

**Background:**

The burden of drug resistant tuberculosis in Africa is largely driven by the emergence and spread of multidrug resistant (MDR) and extensively drug resistant (XDR) *Mycobacterium tuberculosis* strains. MDR-TB is defined as resistance to isoniazid and rifampicin, while XDR-TB is defined as MDR-TB with added resistance to any of the second line injectable drugs and any fluoroquinolone.

The highest burden of drug resistant TB is seen in countries further experiencing an HIV epidemic. The molecular mechanisms of drug resistance as well as the evolution of drug resistant TB strains have been widely studied using various genotyping tools. The study aimed to analyse the drug resistant lineages in circulation and transmission dynamics of these lineages in Africa by describing outbreaks, nosocomial transmission and migration. Viewed as a whole, this can give a better insight into the transmission dynamics of drug resistant TB in Africa.

**Methods:**

A systematic review was performed on peer reviewed original research extracted from PubMed reporting on the lineages associated with drug resistant TB from African countries, and their association with outbreaks, nosocomial transmission and migration. The search terms “Tuberculosis AND drug resistance AND Africa AND (spoligotyping OR molecular epidemiology OR IS*6110* OR MIRU OR DNA fingerprinting OR RFLP OR VNTR OR WGS)” were used to identify relevant articles reporting the molecular epidemiology of drug resistant TB in Africa.

**Results:**

Diverse genotypes are associated with drug resistant TB in Africa, with variations in strain predominance within the continent. Lineage 4 predominates across Africa demonstrating the ability of “modern strains” to adapt and spread easily. Most studies under review reported primary drug resistance as the predominant type of transmission. Drug resistant TB strains are associated with community and nosocomial outbreaks involving MDR- and XDR-TB strains. The under-use of molecular epidemiological tools is of concern, resulting in gaps in knowledge of the transmission dynamics of drug resistant TB on the continent.

**Conclusions:**

Genetic diversity of *M. tuberculosis* strains has been demonstrated across Africa implying that diverse genotypes are driving the epidemiology of drug resistant TB across the continent.

## Background

Multidrug resistant tuberculosis (MDR-TB) is defined as resistance to isoniazid and rifampicin, the most potent anti-TB drugs, while extensively drug resistant tuberculosis (XDR-TB) is defined as MDR-TB with additional resistance to any of the second line injectable drugs (aminoglycosides) and any fluoroquinolone (FQ) [1, 2]. Rifampicin resistance (RR) is used as a proxy for MDR-TB and rapid detection of RR strains is recommended [1, 2].

### Burden of drug resistant tuberculosis in Africa

Globally, an estimated 10 million people developed TB in 2017 alone with over half a million estimated RR-TB cases (82% of which had MDR-TB) [1]. Close to 50% of MDR/RR-TB cases were reported in three countries, namely; India, China and Russian Federation. In 2017, 26,845 MDR/RR-TB and 867 XDR-TB cases were notified in Africa [1]. Of the notified MDR/RR- and XDR-TB cases, treatment enrolment was significantly low (21% for MDR/RR-TB and 1% for XDR-TB) [1]. The highest proportion of TB/HIV co-infection is also seen in this continent (31% on average), with some regions having co-infection rates higher than 50% [1, 3]. It is therefore important to identify TB/HIV co-morbidity in these high risk areas.

### Treatment regimens implemented

Up to 2018, the World Health Organisation (WHO) recommended that MDR-TB be treated with a standard regimen of second line anti-TB drugs which includes a combination of an injectable drug, a fluoroquinolone, other core anti-TB agents as well as the first line anti-TB drugs pyrazinamide and ethambutol, subject to drug susceptibility testing (DST) results [2]. These drugs are however less potent, more toxic and require a prolonged treatment period of up to 24 months. More recently however, the WHO has endorsed a shorter 9–12 month regimen which has been demonstrated to be equally effective in the treatment of MDR-TB and consists of a combination of anti-TB agents [3, 4]. Since 2014, at least 12 countries have introduced this short MDR-TB regimen in Africa [4]. Inappropriate implementation of the shorter MDR-TB treatment regimen however poses a risk of acquiring additional resistance in affected patients, as currently observed for the longer MDR-TB treatment regimen [3, 4]. It is in this light that the WHO recommends DST before commencement of treatment and that the shorter regimen only be made available to patients that have not received prior MDR-TB treatment [4]. Furthermore, the shorter MDR-TB regimen is not recommended for patients with second-line drug resistance, pregnant patients and patients with extrapulmonary TB [4].

### Diagnosis of drug resistant tuberculosis

Culture-based phenotypic DST (pDST) remains the gold standard for the diagnosis of drug resistant TB [1]. The WHO has however endorsed the use of nucleic acid tests (NATs) such as the GeneXpert MTB/RIF assay and the molecular line probe assay (LPA), which provide a more rapid diagnosis [1]. However, they are limited in the range of drug susceptibility that can be detected [1]. Furthermore, the running costs associated with these techniques, the need for expertise and the lack of availability at point of care could explain the low uptake of these rapid diagnostic tools across Africa.

The diagnostic algorithm for drug resistant TB varies across Africa with 15 out of 25 high TB and high MDR-TB burden countries being listed as having a national policy that recommends the use of rapid diagnostic tools as the initial diagnostic tool for presumptive TB [1]. Furthermore 12 out of 25 high TB and high MDR-TB burden countries in Africa are reported as having a national policy for universal pDST [1]. However the number of cases tested with rapid diagnostic tests and pDST is highly variable, with largely poor diagnostic coverage, demonstrating that a high proportion of drug resistant cases go undetected. Of concern is the low rate of DST results for rifampicin and second line drugs. Overall, there is a need to strengthen laboratory capacity and to increase uptake of rapid diagnostic tools in order to improve case detection and treatment of drug resistant TB in Africa.

### Drug resistance tuberculosis surveillance

Routine and frequent epidemiological surveillance is critical for understanding the burden of drug resistant TB in a given region and for planning and policy development and policy implementation. The major drug resistance TB surveillance methods that have been used in Africa include case notifications combined with expert opinions, prevalence surveys, and capture-recapture to estimate incidence [1]. However, the most effective drug resistance monitoring tool has been demonstrated to be continuous surveillance of TB patients through pDST and systematic analysis of routinely collected data [1]. It is a concern that there is scanty data on the prevalence of drug resistant TB across Africa [1].

Between 2010 and 2015, only 16 of 54 African countries (30%) completed national drug resistance prevalence surveys [1]. Older drug resistance survey data is available from 8 countries for the period 2005 and 2009 [1]. Since 2016, there were drug resistance TB surveys on-going in 7 countries while fourteen countries in Africa currently do not have any survey data [1]. From the countries with repeat drug resistance survey data, some countries have reported an increase in the prevalence of MDR-TB and drug resistant TB in general [5, 6]. Other countries have demonstrated no significant changes in prevalence rates of drug resistant TB [7–9].

### Molecular typing tools in epidemiological investigations

Since mid-1990s, several techniques have been validated for use in molecular epidemiological investigations of *M. tuberculosis* strain diversity and clustering including spacer oligonucleotide typing (spoligotyping), insertion sequence *6110*-based restriction fragment length polymorphism (IS*6110*-RFLP) and Mycobacterial Interspersed Repetitive Units – Variable Number Of Tandem Repeats (MIRU-VNTR) [10–12]. Furthermore, next generation whole genome sequencing (WGS) of *M. tuberculosis* clinical isolates provides invaluable knowledge on genetic diversity and microevolution of the *M. tuberculosis* genomes in circulation [13]. Whole genome sequencing is preferred to other typing techniques due to the robustness and high resolution offered by the technique [13]. It however does not negate the usefulness of other typing tools due to limitations experienced in resource limited countries. These include the lack of expertise to set up libraries and to analyse sequencing data, the cost of equipment and the general running cost.

Several epidemiological studies have been conducted across Africa, focused on drug resistance, transmission dynamics and the population structure of drug resistant TB strains [14–16]. However, there is very limited systematic data on the molecular epidemiology of drug resistant TB in Africa. This review therefore aims to synthesise available knowledge of drug resistant TB in Africa, with a particular focus on lineages in circulation, and lineages associated with outbreaks, nosocomial transmission and migration.

## Methods

### Search strategy and selection criteria

A systematic review was conducted of peer reviewed original research on the molecular epidemiology of drug resistant TB from African countries, extracted from PubMed on July 3, 2019 for relevant articles published between 1999 and 2019. The search terms “Tuberculosis AND drug resistance AND Africa AND individual country name for all 54 African countries AND (spoligotyping OR molecular epidemiology OR IS*6110* OR MIRU OR DNA fingerprinting OR RFLP OR VNTR OR WGS)” were used to identify relevant articles reporting the molecular epidemiology of drug resistance in Africa. Studies were eligible for inclusion in the analysis if they described the lineages associated with drug resistant TB, outbreaks, nosocomial transmission and migration in any African countries using one or more of the following techniques; spoligotyping or IS*6110* RFLP or MIRU VNTR or WGS. The search resulted in 187 articles of which 55 met the inclusion criteria, as summarised in Table 1. To generate the review, the following variables were extracted from the studies; pDST, proportion of clustered drug resistant strains, HIV/TB coinfection rate and genotyping methods.

**Table 1:** Genotypes associated with drug resistant TB across Africa

Country	Region (No. of DR samples/total in study)	DST phenotype (% of isolates)	HIV/TB coinfection in DR-TB cases %	Genotype (%)	Genotyping method	Ref.
Angola	Luanda (22/89)	MDR-TB (13.5%) mono-resistant TB (55%), poly-resistant (31.5%)	Reported, but not specified for DR cases.	LAM1 (36%), T1 (23.5%), LAM9 (18%), LAM2 (9%), LAM6 (4.5%), T2 (4.5%), orphan (4.5%)	MIRU-VNTR, Spoligotyping	[17]
Benin	Countrywide (40/100)	Pre-XDR-TB (5%), MDR-TB (25%), S mono resistant-TB (35%), poly-resistant-TB (22.5%), other mono-resistant TB (12.5%)	Reported, but not specified for DR cases	L1 (3%), L2 (22.5%), L3 (3%), L4 (55%), L5 (13%), *M. bovis* (3%)	Spoligotyping	[18] [19]
	Cotonou (17/194)	S mono resistant (100%)	35%	Beijing (100%)	MIRU-VNTR	
Burkina Faso	Ouagadougou (3/58)	MDR-TB (33%), mono-resistant TB (67%)	33%	T (67%), Haarlem (33%)	MIRU-VNTR, Spoligotyping	[20]
CAR	Bangui (53/318)	MDR-TB (100%)	26%	T (47%), proportion of Cameroon, H, EAI not specified	Spoligotyping	[21]
Cameroon	Adamaoua (35/437)	MDR (16%), mono-(71%) & poly-resistant (13%)	Reported, but not specified for DR cases	Cameroon (68.5%), T1 (17%), U (8.5%), H (3%), T2 (3%)	MIRU-VNTR, Spoligotyping	[22]
Chad	Countrywide	MDR-TB (19%) mono-resistant TB (81%)	Not reported	T (5%), Cameroon (60%), H (25%), X (4%), EAI (2%), S (2%), undefined (2%)	MIRU-VNTR, Spoligotyping	[23]
	N’djamena (13/33)	Mono-resistant TB (77%), poly-resistant TB (23%)	Not reported	T (46%), H (31%), H37Rv (8%), EAI (8%), Orphan (7%)	Spoligotyping	[24]
Congo Brazzaville	Brazzavile & Pointe Noire (21/46)	MDR-TB (71%), I mono-resistant (19%), S mono- resistant (5%), IS poly resistant TB (5%)	Not reported	T (67%), Beijing (20%), LAM (13%)	DNA sequencing, MIRU-VNTR	[25]
Djibouti	Countrywide (15/435)	MDR-TB	Not reported	Beijing (73%), T (27%)	MLVA, Spoligotyping, WGS	[26]
	Djibouti city (29/32)	XDR-TB (14%), MDR-TB (79%), mono-resistant TB (7%)	Not reported	CAS (24%), LAM (21%), Orphan (21%), EAI (17%), T (10%), Beijing (3.5%), X (3.5%)	IS*6110*-RFLP, MIRU-VNTR, Spoligotyping	[27]
Egypt	Countrywide (16/67)	Mono-resistant TB (69%), poly-resistant TB (31%)	Not reported	T, LAM, *M. bovis*, CAS, S, undefined	IS*6110*-RFLP, Spoligotyping	[28]
	Assiut (11/25)	MDR-TB (100%)	Not reported	Not defined	IS*6110*-RFLP	[29]
Ethiopia	North-West (116/244)	MDR-TB (10%), mono- & poly- resistant TB (90%),	Reported, but not specified for DR cases	H (32%), T3_ETH (32%), CAS (28%), TUR (2.5%), H37Rv like (2.5%), X (1.5%), Orphan (1.5%)	MIRU-VNTR, Spoligotyping	[30]
	Butajura (95/106)	^a^Poly- (98%), mono-resistant TB (2%)	Reported, but not specified for DR cases	Haarlem (37%), other unspecified	MLPA	[31]
	Jimma (1/15)	I mono resistant (100%)	Reported, but not specified for DR case	T3_ETH	Spoligotyping, DNA sequencing	[32]
	Oromia, SNNRPS, Harari	MDR-TB (15%), mono- & poly-resistant TB (85%)	Not reported	Ethiopia_3 (34%), Lineage 7 (22%), CAS (11%), EA (11%), H37Rv like (7%), H (7%), X (4%), EAI (4%)	Spoligotyping	[33]
Ghana	South-west, Southern and Northern Ghana (71/130)	MDR-TB (6%), mono- & poly-resistant TB (94%)	Not reported	Cameroon (47%), MAF (22%), undefined (31%)	DNA sequencing, IS*6110*-RFLP, Spoligotyping	[34] [35] [36]
Guinea	Conakry (154/359)	MDR-TB (6%), mono- (41%), poly-resistant TB (53%)	Not reported	T (35%), H (20%), CAS (25%), Beijing (10%), S (5%), Orphan (5%)	Spoligotyping	[37]
Kenya	Nairobi (33/73)	MDR-TB (45.5%), poly- (15%), mono-resistant TB (39%)	Not reported	CAS (45.5%), Orphan (30.5%), S (9%), Beijing (6%), LAM (6%), T (3%)	DNA sequencing, Spoligotyping	[38] [39]
	North-Eastern	MDR-TB (14.5%), Mono- (73%), poly resistant TB (12.5%)	Not reported	Not defined	IS*6110*-RFLP, Spoligotyping	
Malawi	Karonga district (116/16870	I resistant (100%)	Reported, but not specified for DR cases	L1 (17%), L3 (18%), L4 (65%)	WGS	[40]
Mali	Bamako (3/20)	XDR (100%)	50%	L4 (100%)	MIRU-VNTR, Spoligotyping	[10]
	Bamako (45/126)	MDR-TB (71%), mono- & poly-resistant (29%)	Reported, but not specified for DR cases	T (64%), MAF2 (11%), LAM (5%), H (5%), EAI (4%), *M. bovis* (3.5%), Beijing (3.5%), other (2%)	Spoligotyping	[41]
Morocco	Casablanca (53/147)	MDR-TB (56%), mono-resistant TB (22%) & poly-resistant (22%)	Not reported	EAI, LAM, H, Beijing, other	MIRU-VNTR	[42]
	Countrywide (19/198)	MDR-TB (37%), Mono- (7%), poly resistant (56%)	Not reported	LAM9 (42%), H (22%), other (21%), Beijing (5%), T (5%), U (5%)	MIRU-VNTR, Spoligotyping	[43]
Mozambique	Countrywide (1/543)	1 MDR-TB case	1 HIV positive case	Beijing	IS*6110*-RFLP, MIRU-VNTR, Spoligotyping	[44]
Nigeria	Cross river state (6/58)	6 MDR-TB cases	33%	LAM10-CAM (83%), T/orphan (17%)	MIRU-VNTR, Spoligotyping	[45]
	Ibadan, Nnewi and Abuja, South-West (29/407)	MDR-TB (76%), mono- & poly-resistant (24%)	Not reported	Cameroon (79%), T (10%), MAF (5%), LAM (3%), U (3%)	MIRU-VNTR, Spoligotyping	[46]
	South-West (36/63)	Pre-XDR- (14%), MDR-TB (86%)	25%	Cameroon (47%), MAF (14%), Ghana (8%), H (8%), LAM (6%), Uganda (6%), H37Rv (6%), X (6%), Orphan (6%)	WGS	[47]
Rwanda	Countrywide (67/151)	MDR-TB (96%), mono-resistant TB (4%)	48%	T2 (72%), Undefined (28%)	RD analysis, Spoligotyping	[48]
Sierra Leone	Western area & kenema district (50/97)	MDR-TB (22%), mono- (48%), poly-resistant TB (30%)	Not reported	Sierra Leone1/2 (26%), LAM (16%), H (16%), MAF (14%), Beijing (8%), S (8%)	IS*6110*-RFLP, MIRU-VNTR, Spoligotyping	[49]
South Africa	Eastern Cape (342/651)	XDR-TB (25%)	Not reported	Beijing (93%), LAM (3%), MANU (3%), S (1%)	DNA sequencing, IS*6110*-RFLP, Spoligotyping	[50] [51]
		Pre- XDR TB (31%)	Not reported	Beijing (92%), LAM (6%), H (1%), Orphan (1%)		
		MDR-TB (44%)	Not reported	Beijing (39%), LAM (30%), T (12%), S (5%), X (2%), H (1%), U (1%), Orphan (10%)		
	Gauteng (672/984)	XDR-TB (9%)	Not reported	Beijing (45%), LAM (41%), T (5%), H (5%), EAI (2%), X (2%)	MIRU-VNTR, Spoligotyping	[52] [53] [54] [55]
		Pre-XDR-TB (5%)	Not reported	LAM (41%), Beijing (27%), H (14%), EAI (14%), S (4%)		
		MDR-TB (73%)		LAM (29%), S (15%), T (14%), H (13%), EAI (12%), Beijing (11%), X (6%)		
		Mono-resistant TB (13%)		Beijing (37%), S (20%), T (16%), EAI (10%), LAM (8%), X (5%), H (4%)		
	KZN (1051/1139)	XDR-TB & Pre-XDR-TB (30)	88%	LAM4 (F15/LAM/KZN) (44%), X (20%), Beijing (11%), EAI (9%), T (6%), LAM3 (3%), S (3%)	DNA sequencing, IS*6110*-RFLP, Spoligotyping, WGS	[14] [56] [57] [55]
		MDR-TB (56%)		LAM4 (F15/LAM/KZN) (40%), S (35%), T (10%), Beijing (6%), CAS (2%), EAI (2%)		
		Mono- & poly- resistant (14%)		LAM (35%), Beijing (30%), T (16%), EAI (8%), X (7%), S (2%), CAS (2%)		
	Limpopo (20/336)	XDR-TB (10%)	Not reported	LAM4 (50%), X1 (50%)	MIRU-VNTR, Spoligotyping	[52]
		Pre-XDR (5%)		Orphan		
		MDR-TB (85%)		Beijing (35%), LAM (18%), EAI1_SOM (12%), S (12%), Orphan (11%), X (6%), T (6%)		
	Mpumalanga (235/336)	XDR-TB (9%)	Not reported	Beijing (29%), EAI (24%), T (14%), S (10%), X (10%), LAM9 (5%), LAM11 (5%), H (3%)	MIRU-VNTR, Spoligotyping	[52]
		Pre-XDR (10%)		EAI (22%), T (18%), Beijing (13%), LAM11 (9%), X (9%), S (4%), LAM9 (4%), LAM4 (4%), H (4%), Orphan (13%)		
		MDR-TB (81%)		EAI (22%), T (20%), Beijing (16%), S (11%), H (5%), LAM9 (5%), LAM11 (3%), LAM3 (3%), X (4%), MANU (2%), LAM4 (1%), Orphan (8%)		
	North-West (31/336)	XDR-TB (3%)	Not reported	EAI	MIRU-VNTR, Spoligotyping	[52]
		Pre-XDR (10%)		EAI1_SOM (67%), Orphan (33%)		
		MDR-TB (87%)		Beijing (37%), T (19%), S (11%), EAI1_SOM (7%), LAM3 (7%), LAM11 (7%), Orphan (18%)		
	Western Cape (611/1682)	XDR-TB (9%)	18%	Beijing (45%), LAM (27%), H (8%), X (6%), other (14%)	DNA sequencing, IS*6110*-RFLP, Spoligotyping	[58] [59] [60] [61] [62]
		Pre- XDR-TB (5%)				
		MDR-TB (35%)				
		Mono- & poly-resistant TB (51%)				
Sudan	Omdurman, Khartoum & Port Sudan (108/235)	MDR-TB (24%), mono resistant TB (76%)	Not reported	CAS1(49%), Beijing (2%), undefined (49%)	MIRU-VNTR, Spoligotyping	[63]
Tanzania	Chagga and Masai tribes (12/111)	MDR-TB (25%), mono- (67%) & poly-resistant TB (8%)	42%	LAM (42%), CAS (17%), T (17%), EAI (8%), MANU (8%), orphan (8%)	Spoligotyping	[64]
Tunisia	Bizerte 21	21 MDR-TB cases	0%	Haarlem3 (95%), undefined (5%)	MIRU-VNTR, Spoligotyping, PCR typing	[65]
Uganda	Mubende district (13/67)	MDR-TB (15%), mono- (69%), poly-resistant TB (16%)	Reported, but not specified for DR case	T (38%), CAS (23%), U (8%), LAM (8%), undefined (23%)	MIRU-VNTR, Spoligotyping, RD analysis	[66]
	Mbabara district (20/125)	MDR-TB (10%), mono- (40%), poly-resistant TB (50%)	Reported, but not specified for DR case	Uganda (45%), CAS (25%), LAM (20%), undefined (10%)	Spoligotyping, RD analysis	[67]
	Kampala district (75/497)	MDR-TB (16%), mono- & poly-resistant TB (84%)	Reported, but not specified for DR case	T (27%), T2-Uganda (18%), CAS (20%), LAM (15%), orphan (12%), undefined (6%)	Spoligotyping	[68]
	Kampala district	MDR-TB (54%), I mono-resistant TB (46%)	29%	T (71%), LAM9 (11%), Uganda (3.5%), Beijing (3.5%), orphan (11%)	Spoligotyping	[69]
Zimbabwe	Countrywide (58/86)	Pre-XDR (27%), MDR-TB (73%)	Not reported	LAM11_ZWE (28%), LAM other (29%), T (16%), Beijing (13%), CAS (5.5%), S (5.5%), MANU (3%)	Spoligotyping	[70]

^a^Based on genotyping. *Abbreviations*: *XDR-TB* Extensively drug resistant tuberculosis, *MDR-TB* Multidrug resistant tuberculosis, *R* Rifampicin, *H* Isoniazid, *E* Ethambutol, *S* Streptomycin, *WGS* Whole genome sequencing, *MLVA Multiple loci VNTR analysis, IS6110-RFLP* Insertion Sequence *6110*-Restriction Fragment Length Polymorphism, Spoligotyping Spacer oligonucleotide typing, *MIRU-VNTR* Mycobacterial interspaced repeat units-variable number of tandem repeats, *PCR* Polymerase Chain Reaction, *CAS* Central Asian, *EAI_SOM* East African Indian_Somalia, *KZN* KwaZulu-Natal, *LAM* Latin American Mediterranean, *MAF Mycobacterium africanum*, *H* Haarlem, *ETH* Ethiopia, *SNNRPS* Southern Nations Nationalists and Peoples Regional State‚ *ref* reference

## Results

### Overview of drug resistant *Mycobacterium tuberculosis* strain types in Africa

#### Molecular epidemiological data

The molecular mechanisms of drug resistance as well as the evolution of drug resistant strains in Africa have been studied using a variety of genotyping tools [10–13]. This has provided some insight into the transmission dynamics of drug resistant TB. Most studies (89%) under review here have used spoligotyping to describe the molecular epidemiology of drug resistant TB in Africa although there are a number of studies which have used highly discriminatory methods which include WGS, IS*6110*-RFLP and MIRU-VNTR [13–16].

#### Population structure of drug resistant TB genotypes in Africa

**Fig. 1:**
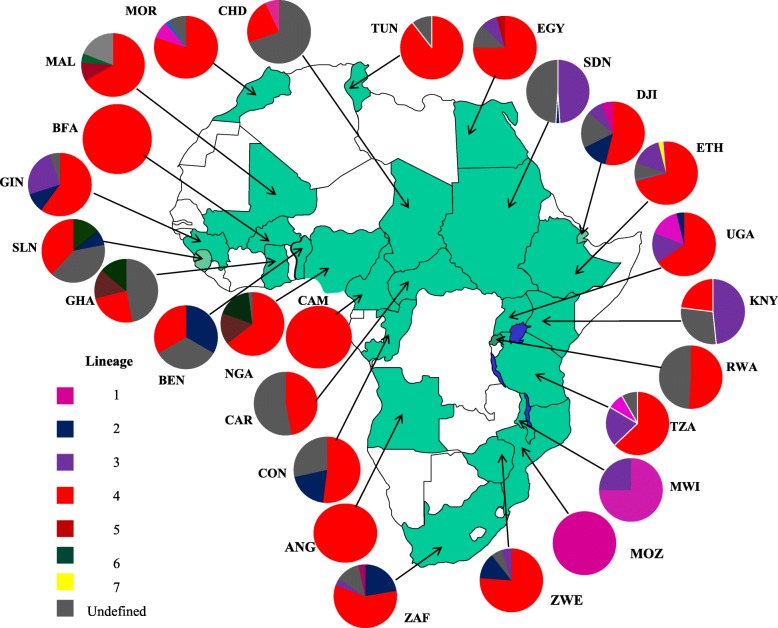
Distribution of *M. tuberculosis* strains according to the 7 major lineages. Varying genotyping tools were used to characterise isolates including spoligotyping, MIRU-VNTR, PCR typing, and WGS, further described in Table 1. *Note*: Figure generated from references listed in Table 1. Countries highlighted in green are countries with published data on the molecular epidemiology of drug resistant TB in Africa

**Fig. 2:**
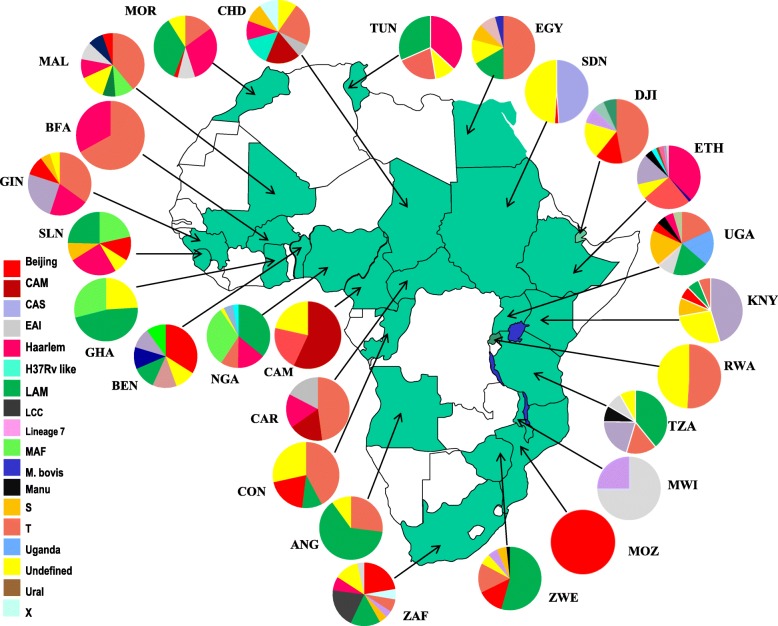
Genotypic distribution of drug resistant *M. tuberculosis* isolates characterised across Africa; largely based on spoligotyping. *Note:* Figure generated from references listed in Table 1. Countries highlighted in green are countries with published data on the molecular epidemiology of drug resistant TB

Sporadic molecular mycobacteriological studies have been conducted within Africa (Figs. 1 and 2), with South Africa having the vast majority of data on the continent. Diverse genotypes have been associated with drug resistant TB (Fig. 1, Fig. 2, Table 1), with particular genotypes being more predominant [52, 58, 59, 66, 71]. For instance, the Beijing genotype is widespread across parts of Africa [38, 44, 60]. The population structure of drug resistant TB is however not homogeneous (Figs. 1 and 2), with certain strains being more predominant in specific population groups [26, 38, 53, 72, 73]. For example, the Haarlem and CAS genotypes are predominantly associated with drug resistance including MDR-TB in parts of North and East Africa while in Southern and West Africa the Beijing and LAM genotypes are highly associated with drug resistance (Figs. 1 and 2) [28, 30, 34, 45, 61, 65, 72]. Further, country-wise comparisons show a correlation between genotypes associated with drug susceptible TB and drug resistant TB, implying that drug resistant TB is to a large extent acquired by individuals within their respective African countries [14, 16, 45, 66, 74].

Associations between specific drug resistant TB strains and HIV co-infection have been noted, with high mortality rates being observed in the context of TB/HIV co-infection [56, 64, 74]. Genotypes such as Beijing, Haarlem and LAM have been associated with high levels of drug resistance and high mortality rates in both HIV seropositive and seronegative individuals [50, 51, 57, 65]. A clear distinction has been observed in the population structure of genotypes associated with mono-resistance, MDR- and XDR-TB (Table 1). In parts of South Africa the F15/LAM4/KZN and Beijing genotypes have been associated with XDR-TB while LAM11_ZWE is associated with MDR-TB in parts of Zimbabwe [54, 61, 70].

A high degree of clustering of drug resistant TB isolates has been observed in parts of Africa [23, 39, 40, 75]; this is of great concern as it implies that there is recent and ongoing transmission of drug resistant TB strains within the region. Furthermore, a correlation between drug resistant strains in the adult population and in children has been demonstrated [62], suggestive of adult to child transmission. There is however very limited molecular typing data on drug resistant TB amongst children and household contacts of drug resistant TB patients in the rest of Africa to confirm this.

Modern lineages (East Asian, EAI and Euro American) have been associated with drug resistance in Central and West Africa (Figs. 1 and 2) [18, 21], regions predominantly associated with *Mycobacterium africanum* (MAF) [18, 21, 35, 37]. Lineage 5 (West-Africa 1) and 6 (West-Africa 2) however continue to predominate in West Africa and are largely associated with drug susceptible TB [24, 36, 46, 49]. The introduction of these drug resistant “modern strains” threatens management of drug resistant TB in the region [22, 31, 67, 68, 76].

### Application of molecular methods to describe transmission dynamics of drug resistant tuberculosis in Africa

#### Acquired MDR- and XDR-TB

There is evidence that acquisition of MDR-and XDR-TB also plays an important role in the burden of drug resistant TB in endemic regions of Africa [77–81]. Inadequate treatment has been shown to be a significant driving force in the development of drug resistant TB, driven by factors such as poor adherence to treatment, diagnosis delay and low quality anti-TB drugs [82, 83]. The severity of drug resistance in South Africa has been demonstrated to be much higher than other parts of Africa, this could be related to South Africa being the first country to administer second-line treatment on the continent in 2001 [84], and could be also be related to better reporting in South Africa.

The WHO recommends the use of a standardized TB treatment regimen which has been adopted by most countries in the region [2]. In the absence of laboratory monitoring and surveillance, mainly due to poor infrastructure and lack of resources, the risk of acquiring resistance is heightened in high TB burden settings [19, 82, 85]. Further, standardized TB treatment has been shown to be unsuccessful in preventing the spread of drug resistant TB [83, 86]. Therefore, there is a need to implement routine DST and surveillance, supported by molecular epidemiology, for active case finding and to guide effective TB treatment in high risk population groups. On the contrary, a standardized shorter MDR-TB regimen has been demonstrated to be highly effective, with a treatment success rate of 89% in Cameroon, a high MDR-TB setting [87].

#### Outbreaks

Drug resistant strains of *M .tuberculosis* have been linked with six distinct outbreaks in parts of Africa (Table 2) [19, 56, 59, 60, 65, 82]. Outbreaks are characterised by sporadic spread of a particular strain of drug resistant TB unlike ongoing transmission which is characterised by constant spread of strains over a longer period of time. A prominent outbreak in Tugela Ferry KZN (mostly amongst HIV positive individuals) involving the F15/LAM4/KZN lineage, brought global focus onto XDR-TB and revealed that XDR-TB strains are transmissible [56]. The main factors associated with the outbreak were an inadequate TB control program coupled with a high HIV prevalence in the affected population [56]. This stresses the need for improved TB infection prevention and control (IPC) measures, together with rapid diagnostics in the successful control of TB in general and XDR-TB in particular.

**Table 2:** Drug resistant TB genotypes associated with nosocomial transmission and outbreaks across Africa

Country (region)	MTB phenotype (number of cases)	MTB lineage (clustered/total isolates)	Transmission dynamics (nosocomial and/or outbreak)	HIV/TB coinfection^**a**^ (%)	Genotyping method	Ref.
Benin (Cotonou)	S mono-resistant TB (17)	Lineage 2/Beijing (17/194)	Community outbreak	6/17 (35%)	MIRU-VNTR	[19]
Mali (Bamako)	XDR-TB(3)	Lineage 4 (3)	Nosocomial transmission	1/2 (50%)	MIRU-VNTR, Spoligotyping	[15]
South Africa (KZN)	XDR-TB (148)	Lineage 4 (53/148)	Nosocomial transmission	123/126 (98%)	DNA sequencing, IS*6110*-RFLP, Spoligotyping	[88]
South Africa (KZN)	MDR-TB (3)	Lineage 4 /F15/LAM4/KZN (3/3)	Nosocomial transmission	HIV status of clustered isolates not defined	IS*6110*-RFLP	[89]
South Africa (North-Western)	I mono-resistant TB (13/128) Poly-resistant TB (7/128) MDR-TB (108/128) Pre-XDR-TB (26/108) XDR-TB (5/108)	Lineage Not specified (74/128)	Community outbreak and nosocomial transmission	84/91 (92%)	DNA sequencing, IS*6110*-RFLP, MIRU-VNTR, Spoligotyping	[82]
South Africa (Western Cape)	MDR-TB (209)	L2/Beijing (62/209)	Community outbreak	Not specified	DNA sequencing, IS*6110*-RFLP, MIRU-VNTR, Spoligotyping	[59]
South Africa (Western Cape)	MDR-TB (21)	L2/Beijing (16/21)	Community outbreak	0%	IS*6110*-RFLP	[60]
Tunisia	MDR-TB (21)	Lineage 4/Haarlem3 (19/21)	Community outbreak	0%	IS*6110*-RFLP, Spoligotyping	[65]

^**a**^Only cases with a known HIV status were included in the analysis. *Abbreviations*: *H* Haarlem, *I* Isoniazid, *IS6110-RFLP* Insertion Sequence *6110*-Restriction Fragment Length Polymorphism, *KZN* KwaZulu-Natal, *MDR-TB* Multidrug resistant tuberculosis, *MIRU-VNTR* Mycobacterial interspaced repeat units-variable number of tandem repeats, *MTB Mycobacterium tuberculosis*, *R* Rifampicin, *ref*. reference, *S* Streptomycin, *Spoligotyping* Spacer oligonucleotide typing, *XDR-TB* Extensively drug resistant tuberculosis

Outbreaks in vulnerable population groups of institutionalized and HIV positive individuals have also been documented [56, 82]. High clustering rates of drug resistant isolates were observed in a mining community which had a high rate of HIV sero-positive individuals (Table 2) [82]. The outbreak was as a result of an inefficient TB control program and diagnosis delay with the biannual chest radiography screening only diagnosing 30% of TB cases in this group of miners [82]. Recommendations have since been made to improve detection and to promote parallel treatment of TB and HIV in high risk groups [82].

Community outbreaks of MDR-TB in HIV sero-negative, non-institutionalized individuals have also been reported [19, 60]. Molecular investigations have revealed diversity in genotypes associated with outbreaks of drug resistant TB (Table 2). Genotypes initially identified to be responsible for drug resistant TB outbreaks have been demonstrated to re-emerge in communities as was the case in Tunisia [90]. A subsequent MDR-TB Haarlem strain outbreak was reported amongst the post-outbreak patients’ population group in which the same strain was identified as the progenitor [90]. The findings of these drug resistant TB outbreak studies emphasise that MDR-TB and indeed other drug resistant TB outbreaks are not limited to specific population groups such as the immunocompromised and the institutionalized [60, 65, 90].

There is some evidence that particular bacterial genotypes are associated with outbreaks. The Beijing genotype for instance, which is endemic in parts of South Africa, was linked to an outbreak of MDR-TB at a school in the Western Cape Province [59]. Molecular characterization confirmed that all isolates belonged to cluster R220 [59]. The genotype was further associated with a streptomycin-resistant outbreak in Benin (Table 2) [19]. The occurrence of an outbreak caused by the Beijing genotype in West Africa further highlights the regional emergence of “modern strains” which appear highly virulent and pose a potential threat to TB control efforts in the region.

While host and strain genetics may play a role in driving outbreaks, inappropriate treatment, non-compliance to treatment and delays in diagnosis are amongst risk factors that have been linked to outbreaks within the continent [56, 60, 82].

#### Nosocomial transmission

The extremely limited data on nosocomial transmission of drug resistant TB in Africa is alarming and places emphasis on the need for molecular epidemiological studies in these high risk settings. Hospital-acquired drug resistant TB has been reported in Africa (Table 2) [15, 82, 88, 89]. An outbreak of the XDR-TB F15/LAM4/KZN strain was described in a district hospital in Tugela Ferry, KZN, South Africa [88]. Epidemiological links for 82% of the patients were made and clustering was observed in 92% of strains [88]. The major risk factors that have been associated with hospital-acquired drug resistant TB are lack of proper IPC measures such as overcrowded wards, poor ventilation and delayed diagnosis [15, 88]. This coupled with the high HIV prevalence experienced in most TB endemic regions makes nosocomial transmission a significant driving force in the transmission of drug resistant TB strains.

Rather than a single point-source outbreak, social network analysis has revealed that patients linked to nosocomial transmissions have a high degree of community interconnectedness [82, 88, 91]. This implies that transmission is occurring both in the community and in the health care facilities (Table 2). Prolonged exposure to patients with drug resistant TB and frequent, concurrent hospital admissions were common in most XDR-TB patients providing strong evidence that nosocomial transmission had occurred [88, 91].

Transmission of TB and drug resistant TB in particular is not only limited to patients receiving care and treatment in health care facilities but has been described in healthcare workers (HCWs) [92]. HCWs are at an increased risk of acquiring drug resistant TB at the work place, especially in the absence of effective IPC measures [93]. It has been demonstrated that diabetes mellitus and HIV infection are common co-morbidities in HCWs that were infected with MDR-TB in a teaching hospital in South Africa [92]. Other factors that have been associated with occupational acquisition of drug resistant TB and TB in general include: increased contact with patients who typically present to the health care facility when they are highly infectious, complacency and low awareness of self-risk typically seen in longer-serving HCWs [92, 93].

Recommendations made towards improved control measures are to prevent transmission through early diagnosis of resistant TB, minimize congregation areas in hospitals by redesigning wards and out-patient areas and use of personal protective equipment [89, 91–93].

#### Migration

Migration has been demonstrated to play a critical role in the spread of drug resistant TB strains globally, with the majority of cases being reported in high-income countries originating from economic migrants from high TB burden countries [94]. There is abundant literature from high-income countries owing to excellent TB surveillance and monitoring [94]. In Africa however, there is very limited information on the impact of migration on transmission of drug resistant TB; this is mainly due to poor surveillance and monitoring. Further, migrant populations typically have poor access to health care and social structures.

Lineages and strains that had previously not been described in particular population groups have been hypothesised to have been introduced to various regions by immigrants [39, 86, 94]. However, the absence of baseline data makes it rather difficult to prove this hypothesis as there is very limited data on drug resistant genotypes that are in circulation within Africa. On the other hand, migration is rife in Africa, mainly due to political instability, civil wars and poverty, and it poses a major concern in the fight against TB and drug resistant TB in particular [95, 96].

Drug resistant strains with streptomycin resistance were detected in a refugee camp in Kenya [39]. Upon comparison to strains in the general populace, the refugee strains were unique to the camp [39]. The nomadic nature of refugees means that they are highly capable of spreading drug resistant strains [95]. There is a higher possibility of refugees failing to complete treatment due to their drifting nature and instability. Further, there is a possibility that the transmission of drug resistant strains is facilitated by a poor TB control program in the country of origin and/or in the refugee camp [39, 87, 95, 97].

Migration is not only an important factor in transmission of drug resistant TB across country borders and across continents, it has also been demonstrated to be an important means of transmission within countries as a result of movement to new cities and provinces in search of better employment opportunities and better health care facilities [39, 53]. For instance, the F15/LAM4/KZN strain has been shown to be widespread both in districts of KZN and in surrounding areas [53, 98]. Further, transmission of drug resistant TB strains has been demonstrated between provinces and districts in South Africa [99, 100]. This stresses a need for rigorous screening of migrants coming from high TB endemic regions and also calls for development and implementation of TB IPC polices in congregate settings in high TB burden regions. However, the above mentioned recommendations are currently not feasible in most African countries due to the porosity of the borders; therefore it is recommended that employers be more vigilant with screening of migrant workers.

## Discussion

The emergence and spread of drug resistant TB strains in the form of MDR-and XDR-TB continue to hinder global efforts to curb the disease; such as the WHO End TB Strategy which aims to reduce deaths associated with TB as well as cut down on new TB cases [1]. The application of molecular epidemiological tools has enabled a better understanding of the global phylogeography of TB [13–16]. In Africa however, there is very limited and sporadic data for the genotypes associated with drug resistant TB. It is important for African countries to implement rigorous drug resistant TB surveillance systems for early case detection and treatment as well as monitoring of drug resistance trends. Routine surveillance would better inform TB control programs on the incidence of drug resistant TB in a given population.

Knowledge of the genotypes in circulation within a given population and the transmission dynamics of drug resistant TB would be important in guiding policy makers on the efficacy of the current treatment regimen and will help identify deficiencies in national TB control programs. Most studies under review used spoligotyping which offers a low resolution of clusters. Overall, WGS provides a superior level of understanding strain relatedness compared to IS*6110*-RFLP and spoligotyping. There is an urgent need to build in-country capacity to enable molecular investigations to be conducted locally using more advanced techniques of WGS. This would require laboratory capacity and training of laboratory and research personnel and would further require local and international funding.

Genetic diversity of *M .tuberculosis* strains has been demonstrated across Africa implying that diverse genotypes are driving the epidemiology of drug resistant TB across the continent. There are variations from region to region and particular genotypes have been demonstrated to be more predominant in certain countries and regions. There is a high degree of genetic diversity in the predominant strains in West Africa with both ancient and modern strains being associated with drug resistant TB [10, 20, 37, 45].

The Beijing and LAM genotypes are widespread across Africa demonstrating the ability of these “modern strains” to adapt and spread easily [17, 38, 54, 60]. It is however worth noting that the strain relatedness or transmission dynamics of these genotypes are not fully understood due to the lack of highly discriminatory tools of WGS in the reviewed studies. In contrast, the “ancient strains” such as MAF strains are largely restricted to West Africa where these strains are mostly associated with drug susceptible TB [10, 45, 46]. A similar observation is made with the Haarlem genotype which is associated with drug resistant TB in East and North Africa [26, 65].

The drug resistant TB epidemic in Africa has been attributed to several drivers, including socio-economic factors (poverty, overcrowded living conditions) and inefficient TB IPC policies (inappropriate treatment, lack of surveillance, diagnostic and treatment delay). MDR-TB case finding and treatment remain a challenge in Africa with high TB and high MDR-TB burden countries falling short on treatment enrolment of new MDR-TB cases, mainly due to the lack of adequate DST [1]. This highlights the urgent need for development and implementation of TB IPC policies in high-risk population groups and also calls for strengthening of outbreak response measures.

There remains a large pool of MDR- and XDR-TB cases that are untreated and are a potential source of drug resistant TB in the various communities [1]. There is a need for united efforts from the continent to improve case detection and treatment for prevention and control of drug resistant TB. Further, high mortality rates have been observed in MDR- and XDR-TB patients and this is worsened by co-infection with HIV [56]. This places emphasis on the need to strengthen the integration of HIV/TB screening and treatment in Africa.

The main challenge for TB activities across the continent is the lack of adequate funding. The majority of countries receive limited funding toward the national TB program with almost a third of the budget being unfunded on average in Africa [1]. Addressing this shortcoming will require collaborative efforts from global funders as well as domestic support from local government. Concerns regarding international funding increased following the proposed budget cuts after the election of Donald Trump as the president of the USA and after the” Brexit” vote in the UK [101, 102]. Changes from the major global TB funders could result in the disintegration of already weak TB control programs in developing countries across the world.

Political instability is a source for concern as it leads to failing of health care infrastructure which in turn results in poor surveillance and treatment efforts. This has been demonstrated in migrant population groups with high rates of untreated drug resistant TB being found in these groups [94]. There is a need to develop and implement rigorous TB screening and treatment of migrants and TB suspects across Africa. This is however made difficult by the poor laboratory infrastructure such as lack of rapid diagnostic techniques for these highly mobile population groups.

## Conclusions

Through molecular epidemiology, it has been demonstrated that drug resistant TB which is endemic in parts of Africa is both acquired and transmitted. Acquired drug resistant TB is largely driven by inadequate treatment, as seen in the case of standardized treatment in the absence of DST results, and non-adherence to treatment. On the other hand, drug resistant TB has been demonstrated to be transmitted in communities and hospital outbreaks have been reported mainly due to poor IPC measures. On average, the treatment success rates for MDR- and XDR-TB are low for Africa, 54 and 28% respectively.

The gap in knowledge on the transmission dynamics and molecular epidemiology of drug resistant TB across the continent is a hindrance in the management of drug resistant TB and calls for improved surveillance efforts. Molecular epidemiological studies play an important role in understanding the transmission dynamics of drug resistant TB across Africa, and will play a part in addressing this knowledge gap. Addressing these key knowledge gaps will guide effective TB treatment in high risk population groups. Additional studies are required to better understand the epidemiology and associated factors of drug resistant TB in Africa as a whole.

## Data Availability

All data generated or analysed during this study are included in this published article, refer to Table 1.

## Abbreviations

CAM: Cameroon
CAR: Central African Republic
CAS: Central Asian
pDST: Phenotypic drug susceptibility testing
E: Ethambutol
EAI: East African Indian
EAI1_SOM: East African Indian_Somalia
ETH: Ethiopia
FQ: Fluoroquinolone
H or INH: Isoniazid
Km: Kanamycin
KZN: KwaZulu-Natal
LAM: Latin American Mediterranean
LCC: Low copy clade
MAF: *Mycobacterium africanum.*
LPA: Line probe assay
MDR: Multidrug resistant
NATs: Nucleic acid tests
R or RIF: Rifampicin
RFLP: Restriction fragment length polymorphism
RR: Rifampicin resistant
S: Streptomycin
Spoligotyping: Spacer oligonucleotide typing
TB: Tuberculosis
WGS: Whole genome sequencing
WHO: World Health Organisation
XDR: Extensively drug resistant
Z: Pyrazinamide
